# Comparison of DNA methylation levels of repetitive loci during bovine development

**DOI:** 10.1186/1753-6561-5-S4-S3

**Published:** 2011-06-03

**Authors:** Masahiro Kaneda, Satoshi Akagi, Shinya Watanabe, Takashi Nagai

**Affiliations:** 1 Reproductive Biology and Technology Research Team, National Institute of Livestock and Grassland Science (NILGS), National Agriculture and Food Research Organization (NARO), 2 Ikenodai, Tsukuba, Ibaraki 305-0901, Japan

## Abstract

**Abstract:**

**Background**

DNA methylation of cytosine residues in CpG dinucleotide controls gene expression and dramatically changes during development. Its pattern is disrupted in cloned animals suggesting incomplete reprogramming during somatic cell nuclear transfer (the first reprogramming). However, the second reprogramming occurs in the germ cells and epigenetic errors in somatic cells of cloned animals should be erased. To analyze the DNA methylation changes on the spermatogenesis of bulls, we measured DNA methylation levels of three repetitive elements in blastocysts, blood and sperm.

**Methods**

DNA from PBLs (peripheral blood leukocytes), sperm and individual IVF (*in vitro* fertilized) and parthenogenetic blastocysts was isolated and bisulfite converted. Three repetitive elements; Satellite I, Satellite II and *art2* sequences were amplified by PCR with specific pairs of primers. The PCR product was then cut by restriction enzymes and analyzed by agarose gel electrophoresis for determining the DNA methylation levels.

**Results**

Both Satellite I and Satellite II sequences were highly methylated in PBLs, whereas hypo-methylated in sperm and blastocysts. The *art2* sequence was half methylated both in PBLs and sperm but less methylated in blastocysts. There was no difference in DNA methylation levels between IVF and parthenogenetic blastocysts.

**Conclusions**

These results suggest that there is a dynamic change of DNA methylation during embryonic development and spermatogenesis in cattle. Satellite I and Satellite II regions are methylated during embryogenesis and then de-methylated during spermatogenesis. However, *art2* sequences are not de-methylated during spermatogenesis, suggesting that this region is not reprogrammed during germ cell development. These results show dynamic changes of DNA methylation levels during bovine embryogenesis, especially genome-wide reprogramming in germ cells.

## Background

DNA methylation is a major physiological modification in mammalian genome. Cytosine residues in CpG dinucleotide pairs are selectively methylated by DNA methyltransferases and these methylation patterns are maintained throughout cell division. DNA methylation alters gene expression patterns in cells and is crucial for normal mammalian development [1,2]. Usually, repetitive elements such as centromeric repeats and transposon sequences are highly methylated and transcriptionally silenced, called a heterochromatic state. The methylation patterns are dramatically changed during embryonic development, from a fertilized egg to a lot of types of differentiated cells. As DNA methylation controls gene expression, some sets of genes are activated/inactivated in particular types of cells during differentiation. Genomic imprinting, which causes parent-of-origin specific gene expression in mammals and X chromosome inactivation, which compensates X chromosome genes dosage between females (XX sex chromosomes) and males (XY sex chromosomes), are controlled by DNA methylation and are crucial for normal mammalian development [3]. Aberrant DNA methylation patterns were observed in many kinds of tumour cells [4].

In mouse germ cells development, it was previously shown that imprinted genes and repetitive elements were de-methylated in primordial germ cells [5] and then re-methylated during spermatogenesis or oogenesis [6]. *De novo* DNA methyltransferases, Dnmt3a and Dnmt3b and Dnmt3-like protein Dnmt3L are responsible for establishing sex-specific DNA methylation patterns both in males and females [7-10]. After fertilization, there is a passive DNA demethylation in the preimplantation embryos depending on DNA replication, however, methylation of imprinted gene escapes this genome-wide demethylation event. *De novo* methylation begins after implantation by Dnmt3a and Dnmt3b, and these methylation patterns are maintained throughout development by the maintenance DNA methyltransferase Dnmt1 [11,12]. However, little is known about the changes of DNA methylation during embryogenesis in cattle. Here we report the dynamic changes of DNA methylation patterns at three repetitive sequences in bovine blastocysts, somatic cells and sperm.

## Methods

### DNA preparation

Blood and frozen sperm samples from six Japanese Black bulls (Wagyu), two Japanese Brown bulls (Aka-ushi) and one Holstein bull were obtained and genomic DNA was extracted by using DNeasy Blood & Tissue Kit (QIAGEN). Sperm DNA was extracted using the lysis buffer with 200mM dithiothreitol (Sigma-Aldrich). DNA from individual blastocysts was isolated as described previously [12]. IVF and PA embryos were produced by a standard method [13].

### DNA methylation analysis

Genomic DNA was bisulfite converted by EpiTect Bisulfite Kits (QIAGEN) according to the manufacturer’s instructions. Three repetitive elements were amplified with specific pairs of primers previously described [14]. The amplified PCR products were then cut by restriction enzymes (Satellite I by *Aci*I, Satellite II by *Acc*II and *art2* by *Taq*I). After digestion, each size of the digested PCR fragments was isolated by 2% agarose gel electrophoresis.

## Results

**Figure 1:**
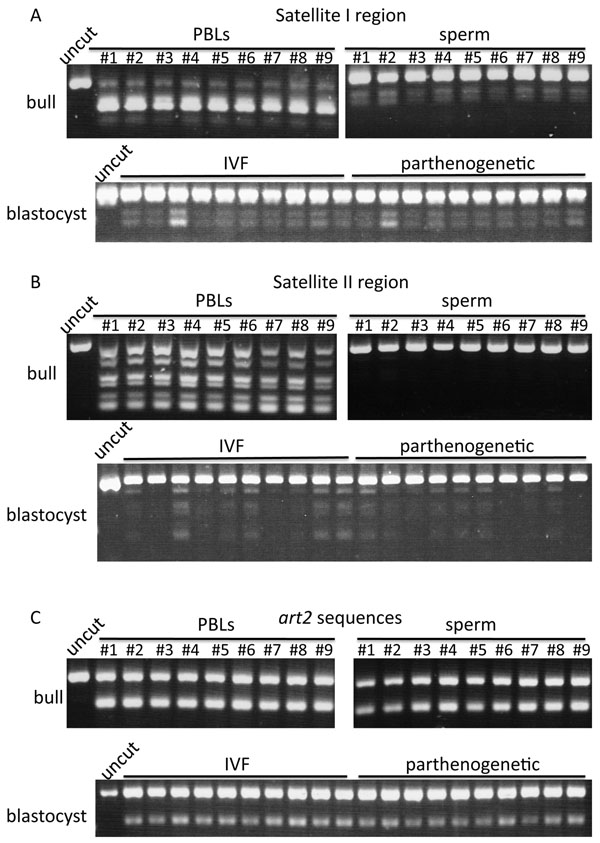
**DNA methylation analysis in PBLs, sperm and blastocysts.** Bulls #1-#9 shows each number of analyzed bulls. Uncut band indicates the PCR product not cut by restriction enzymes. (A) Satellite I regions were cut by *Aci*I. (B) Satellite II regions were cut by *Acc*II. (C) *art2* sequences were cut by *Taq*I.

We analyzed genomic DNA methylation patterns to monitor the changes of epigenetic patterns during bovine embryogenesis and spermatogenesis. We chosen three repetitive regions; Satellite I, Satellite II and *art2* sequences. Satellite sequences are repetitive sequences at the peri-/centromeric regions of the chromosomes, whereas *art2* sequences are *Alu*-like short interspersed nuclear elements (SINEs). First, we analyzed genomic DNA of bull blood (peripheral blood leukocytes, PBLs) and sperm for methylation status of the same regions. We found a large difference in methylation status using restriction enzyme analysis (Figure 1A-C). Satellite I sequences were highly methylated in PBLs (almost PCR fragments were cut by *Aci*I), whereas hypo-methylated in sperm (almost PCR fragments were not cut by *Aci*I) (Figure 1A). Satellite II sequences were also hyper-methylated in PBLs but hypo-methylated in sperm (Figure 1B). However, there were no differences in *art2* sequence methylation levels between PBLs and sperm (Figure 1C). These results clearly indicated that both Satellite I and Satellite II sequences, which are located on the centromeric heterochromatic regions, were de-methylated during spermatogenesis, whereas *art2* sequences, which are located on euchromatic regions, were not methylated/de-methylated during spermatogenesis. Of nine bulls analyzed, there were no differences in DNA methylation patterns of three repetitive elements among individuals and breeds (bulls #1 and #6-9 are Japanese Black, bulls #3-5 are Japanese Brown and Bull #2 is Holstein).

We also analyzed DNA methylation levels in individual blastocysts; 10 *in vitro* fertilized (IVF) and 10 parthenogenetically activated (PA) embryos. Both Satellite I and Satellite II regions were hypo-methylated, whereas *art2* sequences were moderately methylated in IVF and PA blastocysts. There were no differences in DNA methylation patterns between IVF and PA embryos, however, some blastocysts showed more methylated patterns (less cut by restriction enzymes) compared to others (Figure 1A and 1B).

## Discussion

This study shows the DNA methylation changes of repetitive elements during bovine development. The methylation pattern differences of imprinted genes *IGF2* and *SNRPN* in bovine oocytes and sperm have been reported [15,16] and the methylation levels of repetitive elements in bovine blastocysts, especially embryos produced by somatic cell nuclear transfer (SCNT) technology showing high levels of DNA methylation, have also been described [14,17]. However, the DNA methylation status of repetitive elements in sperm DNA has not been well understood. We found that two satellite sequences on the centromeric regions of chromosomes, Satellite I and Satellite II sequences, were highly methylated in PBLs but hypo-methylated in sperm. As these regions were not methylated at blastocyst stages, it is suggested that they are methylated after implantation, and then de-methylated during spermatogenesis. In contrast, *art2* sequences were moderately methylated in blastocysts but more methylated both in PBLs and sperm, suggesting this region is methylated after implantation but not de-methylated during spermatogenesis. There were no differences between IVF and PA blastocysts, suggesting that these repetitive elements are not methylated by a parent-of-origin specific manner. We analyzed total nine bulls with three different breeds; six Japanese Black, two Japanese Brown and one Holstein, however, there were no difference in DNA methylation patterns among individuals. These results suggest that in adults DNA methylation patterns are firmly maintained during embryogenesis and uniformly reprogrammed during spermatogenesis. In contrast, we observed differences of DNA methylation patterns among the individual IVF and PA blastocysts. This could be explained that the variation itself confers the developmental competence of embryos because SCNT embryos with high DNA methylation levels mostly die *in utero* and even IVF embryos, only half of them transferred to the uterus develop to term.

In SCNT embryos, Satellite I, Satellite II regions and *art2* sequences are hyper-methylated compared to IVF embryos [14]. As donor cells also have high DNA methylation levels, it is suggested that the first reprogramming step (transfer donor nucleus to the enucleated oocyte) is not sufficient to fully reprogram the donor genome. SCNT technology has been developed to rewind the differentiation mechanism, however, the efficiency of this artificial reprogramming is quite low so that still now, more than ten years has past since the first cloned sheep Dolly was born, the success rate of SCNT is still less than 5-10% in cattle and other species. This incomplete reprogramming in SCNT and the resulting alternation of DNA methylation and gene expression were described [18]. However, it is hypothesized that the epigenetic errors that were not corrected during the first reprogramming step are erased and then properly reprogrammed (the second reprogramming step) during germ cell development [19]. Therefore, offspring from cloned animals do not show any abnormalities observed in cloned animals themselves. In fact, the obese phenotype frequently observed in cloned mice does not transmitted to the next generation [20]. In cattle, there is no remarkable difference in health status and food products among non-cloned, cloned cattle developed to adulthood and their offspring [21-23]. By applying this study for cloned cattle, it will be possible to prove proper epigenetic reprogramming during cloned cattle gametogenesis and thus contribute to the normality of cloned cattle offspring.

## Competing interests

The authors declare that they have no competing interests.

## Authors' contributions

MK carried out DNA extraction, DNA methylation analysis and analyzed the data. SA made IVF and PA embryos. SW provided blood and sperm samples from bulls. TN participated in the design of the study and contributed to discussion of the results and revision of the paper. All authors read and approved the final manuscript.

## References

[B1] Li E Chromatin modification and epigenetic reprogramming in mammalian development Nature Reviews Genetics 2002 3 662 673 10.1038/nrg887 12209141

[B2] Reik W Stability and flexibility of epigenetic gene regulation in mammalian development Nature 2007 447 425 432 10.1038/nature05918 17522676

[B3] Miyoshi N Barton SC Kaneda M Hajkova P Surani MA The continuing quest to comprehend genomic imprinting Cytogenet Genome Res 2006 113 6 11 10.1159/000090808 16575156

[B4] Jones PA Baylin SB The epigenomics of cancer Cell 2007 128 683 692 10.1016/j.cell.2007.01.029 17320506 PMC3894624

[B5] Hajkova P Erhardt S Lane N Haaf T El-Maarri O Reik W Walter J Surani MA Epigenetic reprogramming in mouse primordial germ cells Mechanisms of development 2002 117 15 23 10.1016/S0925-4773(02)00181-8 12204247

[B6] Lane N Dean W Erhardt S Hajkova P Surani A Walter J Reik W Resistance of IAPs to methylation reprogramming may provide a mechanism for epigenetic inheritance in the mouse Genesis 2003 35 88 93 10.1002/gene.10168 12533790

[B7] Bourc'his D Xu GL Lin CS Bollman B Bestor TH Dnmt3L and the establishment of maternal genomic imprints Science 2001 294 2536 11719692 10.1126/science.1065848

[B8] Kaneda M Okano M Hata K Sado T Tsujimoto N Li E Sasaki H Essential role for de novo DNA methyltransferase Dnmt3a in paternal and maternal imprinting Nature 2004 429 900 903 10.1038/nature02633 15215868

[B9] Bourc'his D Bestor TH Meiotic catastrophe and retrotransposon reactivation in male germ cells lacking Dnmt3L Nature 2004 431 96 99 15318244 10.1038/nature02886

[B10] Kato Y Kaneda M Hata K Kumaki K Hisano M Kohara Y Okano M Li E Nozaki M Sasaki H Role of the Dnmt3 family in de novo methylation of imprinted and repetitive sequences during male germ cell development in the mouse Hum Mol Genet 2007 16 2272 10.1093/hmg/ddm179 17616512

[B11] Okano M Bell DW Haber DA Li E DNA methyltransferases Dnmt3a and Dnmt3b are essential for de novo methylation and mammalian development Cell 1999 99 247 258 10.1016/S0092-8674(00)81656-6 10555141

[B12] Hirasawa R Chiba H Kaneda M Tajima S Li E Jaenisch R Sasaki H Maternal and zygotic Dnmt1 are necessary and sufficient for the maintenance of DNA methylation imprints during preimplantation development Genes Dev 2008 22 1607 1616 10.1101/gad.1667008 18559477 PMC2428059

[B13] Akagi S Hosoe M Matsukawa K Ichikawa A Tanikawa T Takahashi S Culture of Bovine Embryos on a Polydimethylsiloxane (PDMS) Microwell Plate J Reprod Dev 2010 56 475 459 10.1262/jrd.09-213H 20484872

[B14] Kang YK Koo DB Park JS Choi YH Chung AS Lee KK Han YM Aberrant methylation of donor genome in cloned bovine embryos Nature genetics 2001 28 173 177 10.1038/88903 11381267

[B15] Gebert C Wrenzycki C Herrmann D Gröger D Reinhardt R Hajkova P Lucas-Hahn A Carnwath J Lehrach H Niemann H The bovine IGF2 gene is differentially methylated in oocyte and sperm DNA Genomics 2006 88 222 229 10.1016/j.ygeno.2006.03.011 16644179

[B16] Lucifero D Suzuki J Bordignon V Martel J Vigneault C Therrien J Filion F Smith LC Trasler JM Bovine SNRPN methylation imprint in oocytes and day 17 in vitro-produced and somatic cell nuclear transfer embryos Biol Reprod 2006 75 531 538 10.1095/biolreprod.106.051722 16790688

[B17] Kang YK Lee HJ Shim JJ Yeo S Kim SH Koo DB Lee KK Beyhan Z First NL Han YM Varied patterns of DNA methylation change between different satellite regions in bovine preimplantation development Mol Reprod Dev 2005 71 29 35 10.1002/mrd.20249 15736134

[B18] Dean W Santos F Stojkovic M Zakhartchenko V Walter J Wolf E Reik W Conservation of methylation reprogramming in mammalian development: aberrant reprogramming in cloned embryos Proc Natl Acad Sci U S A 2001 98 13734 10.1073/pnas.241522698 11717434 PMC61110

[B19] Fulka J Miyashita N Nagai T Ogura A Do cloned mammals skip a reprogramming step? Nat Biotechnol 2004 22 25 26 10.1038/nbt0104-25 14704696

[B20] Tamashiro KLK Wakayama T Akutsu H Yamazaki Y Lachey JL Wortman MD Seeley RJ D'Alessio DA Woods SC Yanagimachi R Cloned mice have an obese phenotype not transmitted to their offspring Nature Medicine 2002 8 262 267 10.1038/nm0302-262 11875497

[B21] Shiga K Umeki H Shimura H Fujita T Watanabe S Nagai T Growth and fertility of bulls cloned from the somatic cells of an aged and infertile bull Theriogenology 2005 64 334 343 10.1016/j.theriogenology.2004.12.002 15955357

[B22] Watanabe S Nagai T Health status and productive performance of somatic cell cloned cattle and their offspring produced in Japan J Reprod Dev 2008 54 6 1 10.1262/jrd.19090 18319570

[B23] Watanabe S Nagai T Death losses due to stillbirth, neonatal death and diseases in cloned cattle derived from somatic cell nuclear transfer and their progeny: a result of nationwide survey in Japan Anim Sci J 2009 80 233 238 10.1111/j.1740-0929.2009.00640.x 20163630

